# Structural comparison of unconventional G protein YchF with heterotrimeric G protein and small G protein

**DOI:** 10.1080/15592324.2021.2024405

**Published:** 2022-02-08

**Authors:** Maozhen Luo, Zhiwei Han, Guoye Huang, Rongfang Li, Yi Liu, Junjie Lu, Lin Liu, Rui Miao

**Affiliations:** a College of Agriculture, Fujian Agriculture and Forestry University, Fuzhou, China; b College of Life Sciences, Fujian Agriculture and Forestry University, Fuzhou, China

**Keywords:** G proteins, heterotrimeric G proteins, small G proteins, unconventional G proteins, GTP, ATP

## Abstract

Guanine nucleotide-binding (G) proteins, namely, phosphate-binding (P) loop GTPases, play a critical role in life processes among different species. Based on the structural characteristics, G proteins can be divided into heterotrimeric G proteins, small G proteins and multiple unique unconventional G proteins. The highly conserved unconventional G protein YchF is composed of a core G domain, an inserted coiled-coil domain, and a TGS domain from the N-terminus to the C-terminus. In this review, we compared the structural characteristics of the G domain in rice OsYchF1 with those of *Rattus norvegicus* heterotrimeric G protein α-subunit and human small G protein Ras-related G protein C and analyzed the binding modes of these G proteins with GTP or ATP by performing molecular dynamics simulations. In summary, it will provide new insights into the enormous diversity of biological function of G proteins.

Guanine nucleotide-binding (G) proteins, so-called phosphate-binding (P) loop GTPases, regulate multiple aspects of signaling pathways and cellular processes in all kingdoms of life.^1^ On the one hand, G proteins are composed of two large superclasses: SIMIBI (Signal Recognition GTPases and the MinD and BioD) superclass and TRAFAC (Translation Factors) superclass, on the basis of phylogenetic and structural analyses.^2^ Both these can be further subclassified into numerous families and subfamilies.^2^ G proteins in the SIMIBI superclass show seven stranded β-sheet, whereas TRAFAC superclass G proteins are featured by a six stranded β-sheet and one antiparallel strand, with an occasional extra (parallel) strand dependent on binding nucleotide.^2^ G proteins in the TRAFAC superclass contain translation factors, Ras-like GTPases, Obg-related GTPases, and so on. Heterotrimeric G proteins and small G proteins belong to Ras-like GTPases, whereas OsYchF1, a rice YchF, belongs to the YchF subfamily in Obg-related GTPases of the TRAFAC superclass of P-loop GTPases.^2^

On the another hand, according to the structural characteristics, G proteins can also be divided into heterotrimeric G proteins, small G proteins, and multiple unique unconventional G proteins.^3^ OsYchF1 is a kind of unconventional G protein, which comprises three conserved domains, a N-terminal core G domain, flanked by a coiled-coil domain, and a C-terminal TGS (ThrRS, GTPase, and SpoT) domain (Figure 1a). The OsYchF1 G domain displayed a rotated six β-strand sheet as a core, surrounded by a group of α-helices, including a large coiled-coil domain (Figure 1a). The C-terminal TGS domain has a cluster of conserved positive charged amino acid residues (lysine (Lys/K) and arginine (Arg/R)) and is probably involved in nucleic acid binding.^4^

**Figure 1.**
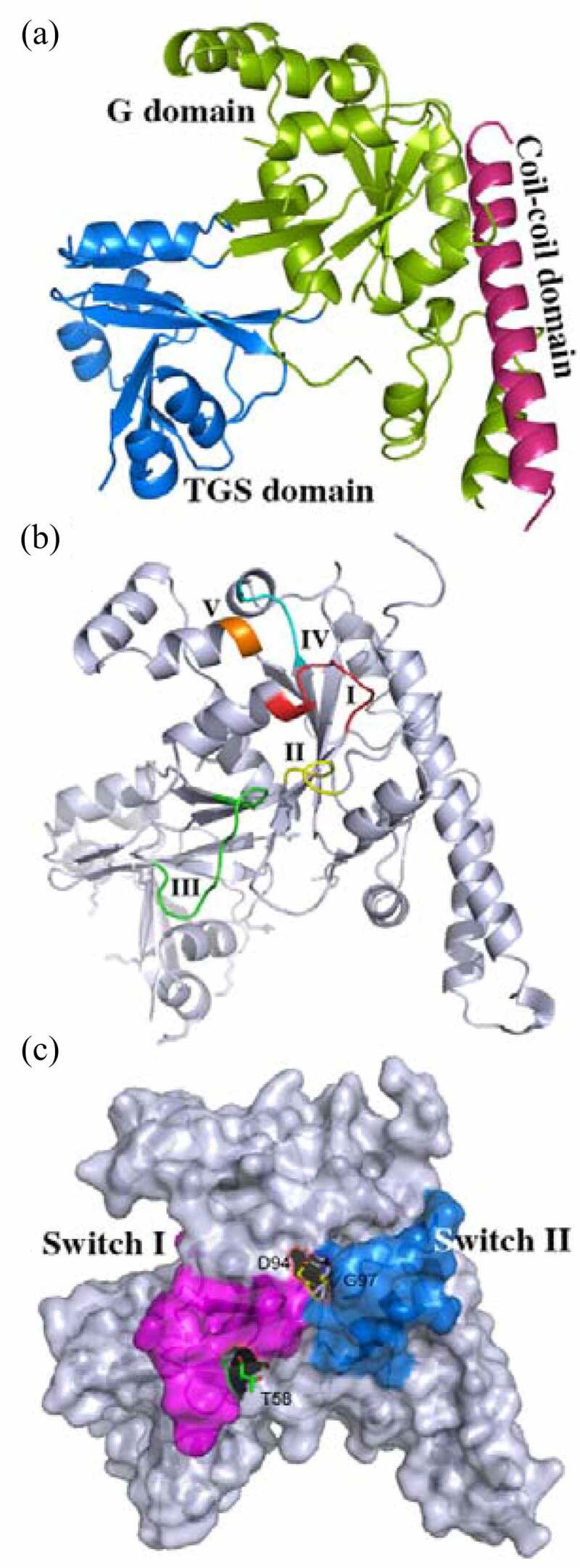
The 3-D structure of nucleotide-free OsYchF1. (a) Cartoon representation of the 3-D structure of OsYchF1 in the nucleotide-free form. The G domain, the TGS domain and the coiled-coil domain are labelled. (b) Cartoon representation of the 3-D structure of OsYchF1, indicating the G1-G5 motif. The G1 motif/P-loop (GxxxxGK(S/ T)), the G2 motif (xT/Sx) the G3 motif (hhhDxxG)the G4 motif ((N/T)(M/L)xD)and the G5 motif ((T/G) (C/S) A) are labelled. (c) Cartoon representation of the 3-D structure of OsYchF1, indicating switch I and II regions. The switch I region with the position of conserved residue threonine (T58), and the switch II region with the position of conserved Asp (D94) and Gly (G97) residues are labelled.

All G proteins possess a core G domain including five conserved fingerprint motifs that range from G1 to G5 motifs. The G1 motif (GxxxxGK(S/T)), namely, P-loop or walk A motif, is associated with the binding for α- and β-phosphate of guanosine triphosphate (GTP). G2 motif (x (T/S) x) and G3 motif (hhhDxxG), namely, walk B motif, bind to the terminal γ-phosphate of GTP. The conserved aspartic acid (Asp/D) residue in the G3 motif (hhhDxxG) binds to the essential cofactor magnesium (Mg^2+^) that is crucial for nucleotide binding and hydrolysis. Mg^2+^ not only connects the β and γ of GTP with walk B motif but also coordinates the conserved threonine (Thr) residue in the G2 motif and moves its backbone carbonyl group into a position with the hydrolytic water to facilitate the nucleophilic assault, resulting in γ-phosphate hydrolysis from GTP.^5^ The walk B motif overlaps with switch I and switch II regions, where conformational switch occurs accompanying with nucleotide hydrolysis; the G4 motif ((N/T)KxD) is critical for the adenosine or guanosine recognition, determining the specific guanine base signature. The G5 motif ((T/G) (C/S) A) supports the specific binding of guanine base, and the backbone of alanine (Ala/A) in the G5 motif forms a hydrogen bond with the oxygen (O-6) in guanine.^6^ The OsYchF1 G domain maintained five conserved motifs as other G proteins. G1, G2, G3, and G5 motifs are conserved and invariant with other G proteins, but the G4 motif ((N/T)KxD) in the YchF subfamily exhibits a unique (N/T)(M/L/V)xE amino acid sequence (Figure 1b). Therefore, the members in the YchF subfamily lose GTP specificity.

The YchF subfamily comprises a group of highly conserved orthologs that share more than 40% sequence identity among different species. The fungus *Schizosaccharomyces pombe* YchF crystal structure (Protein Data Bank (PDB) accession code: 1NI3) was the first solved 3-Dimension (D) structure in the YchF subfamily, and another bacterium *Haemophilus influenzae* YchF was also solved soon (PDB code: 1JAL).^4^ After that, a crystal structure of human YchF ortholog hOLA1 (human Obg-like ATPase 1) (PDB code: 2OHF) was determined. Interestingly, hOLA1 displayed higher ATP binding affinities and hydrolysis rates than GTP.^7^ *Escherichia coli* YchF protein also displayed ATPase priority, while the 70S ribosome binds to and enhances the ATPase activity of YchF (~10 fold).^8^ OsYchF1 crystal structures in the absence or presence of AMPPNP and GMPPNP (PDB code: 5EE0, 5EE3 and 5EE9) are the first solved YchF in plants. Additionally, the OsYchF1 Switch I region includes the G2 motif (xT/Sx), while the G3 motif (DxxG) overlaps with the switch II region in OsYchF1 (Figure 1c).

Herein, heterotrimeric G protein and small G protein, two categories in the TRAFAC superclass, were chosen to compare with OsYchF1 in the 3-D structure (Figure 2a-c). First, the heterotrimeric G proteins exhibit a complex and comprise alpha (α), beta (β), and gamma (γ) subunits.^9^ Beta and gamma subunits bind tightly and form a beta-gamma complex, whereas the α subunit keeps the G domain. The activities of heterotrimeric G proteins are dependent on GTP binding in α subunit. Heterotrimeric G proteins locate intracellularly and are activated by G protein-coupled receptors (GPCRs) that anchor across the plasma membrane to perceive the extracellular signals. The heterotrimeric G proteins function mainly in vision, smell, and taste in animals.^6^ Second, small G proteins are monomeric G proteins with a molecular weight of ~20-40kDa and consists of five families: Ras, Rho, Rab, Ran, and Arf families.

**Figure 2.**
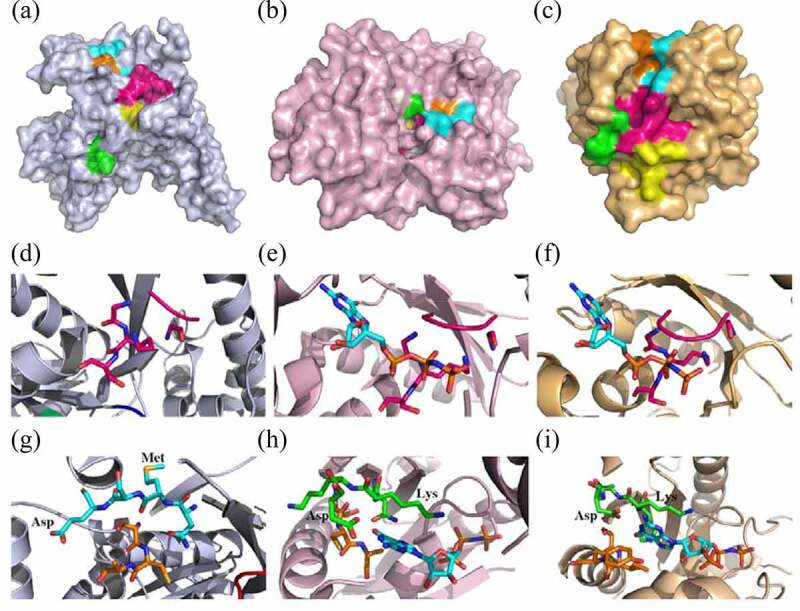
Structural comparison of unconventional G protein OsYchF1 with the *Rattus norvegicus* heterotrimeric G protein α-subunit and small human Ras-related G protein C. (a-c) Cartoon surface representation of the nucleotide-free 3-D structure of OsYchF1, *R. norvegicus* heterotrimeric G protein α-subunit, and human Ras-related G protein C. The G1 motif/P-loop (GxxxxGK(S/T)), the G2 motif (xT/Sx)the G3 motif (hhhDxxG) the G4 motif ((N/T)(M/L)xD), and the G5 motif ((T/G) (C/S) A) re indicated. (d-f) Details of the G1 motif (GxxxxGK(S/T)), P-loop, structure of OsYchF1, *R. norvegicus* heterotrimeric G protein α-subunit with GppNHp that is shown as sticks, and human Ras-related G protein C in the presence of GDP that is shown as sticks. (g-i) Details of G4 ((N/T)KxD) and G5 ((T/G) (C/S) A) motif structure of OsYchF1, *R. norvegicus* heterotrimeric G protein α-subunit with GppNHp that is shown as sticks, and small human Ras-related G protein C in the presence of GDP that is shown as sticks.

Sequence alignments and phylogenetic analysis of the *Rattus norvegicus* heterotrimeric G protein α-subunit (XP_010846404.1), *Oryza sativa* heterotrimeric G protein α-subunit (XP_015639183.1), human Ras-related G protein C (NP_071440.1), *Oryza sativa* Ras-related G protein Rab-2-B (XP_015626284.1), and YchF homolog (BAD03576.1, NP_174346.1, XP_001776504.1, XP_002445191.1, XP_002263885.1, AAF65513.1, XP_002523184.1, and ABK25108.1) were performed by using the Jalview version 2.11.1.3 software (http://www.jalview.org/) (Figures 3 and 4). The phylogenetic tree indicated that the *R. norvegicus* heterotrimeric G protein α-subunit, *Oryza sativa* heterotrimeric G protein α-subunit, human Ras-related G protein C, and *Oryza sativa* Ras-related G protein Rab-2-B are close to each other and stand by themselves, respectively, and displayed low amino acid sequence identities with YchF homolog (Figure 4).

**Figure 3.**
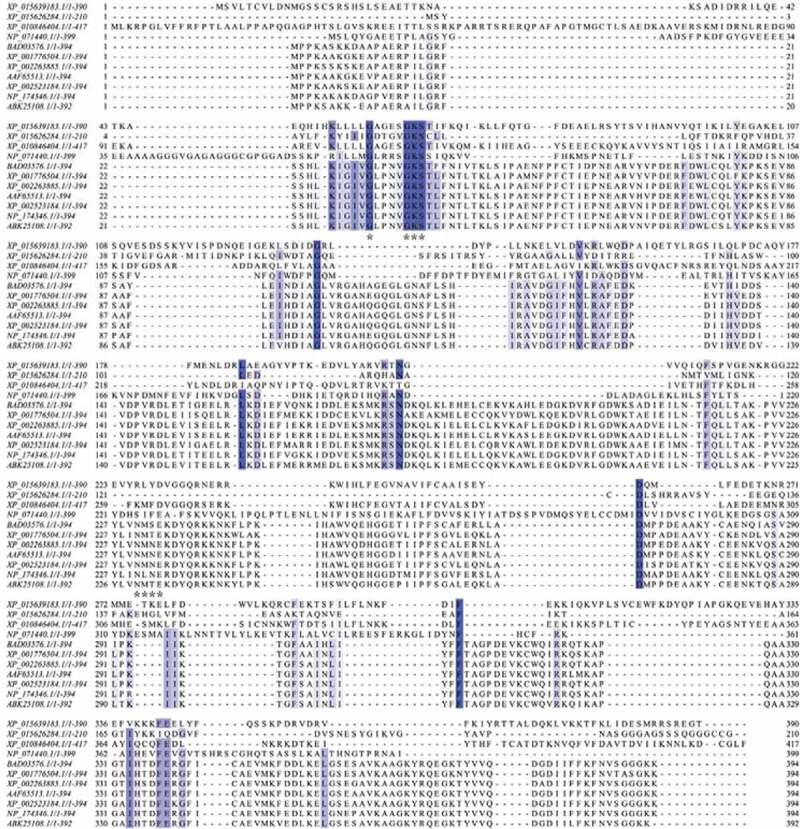
Sequence alignments of the indicated heterotrimeric G protein α-subunit, small G protein, and YchF homolog. Sequence of the *Rattus norvegicus* heterotrimeric G protein α-subunit (XP_010846404.1), *Oryza sativa* heterotrimeric G protein α-subunit (XP_015639183.1), *Homo sapiens* Ras-related G protein C (NP_071440.1), *Oryza sativa* Ras-related G protein Rab-2-B (XP_015626284.1), *Oryza sativa* OsYchF1 (BAD03576.1), *Arabidopsis thaliana* AtYchF1 (NP_174346.1), *Physcomitrella patens* YchF (XP_001776504.1), *Sorghum bicolor* YchF (XP_002445191.1), *Vitis vinifera* YchF (XP_002263885.1), *Capsicum annuum* YchF (AAF65513.1), *Ricinus communis* YchF (XP_002523184.1), and *Picea sitchensis* YchF (ABK25108.1), which were aligned using Jalview version 2.11.1.3 software. Identical amino acid residues are marked in dark. The G1 and G4 motifs of the G domain are marked with asterisks.

**Figure 4.**
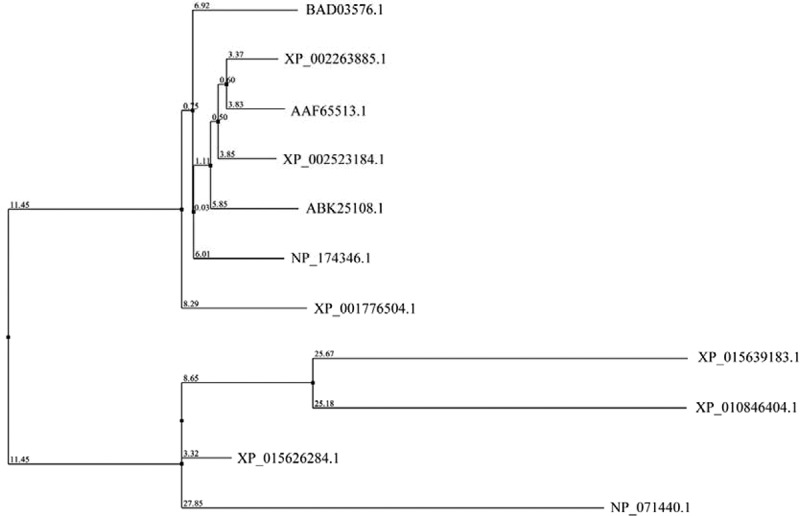
Phylogenetic analysis of the indicated heterotrimeric G protein α-subunit, small G protein, and YchF homolog. *R. norvegicus* heterotrimeric G protein α-subunit (XP_010846404.1), *Oryza sativa* heterotrimeric G protein α-subunit (XP_015639183.1), *Homo sapiens* Ras-related G protein C (NP_071440.1), *Oryza sativa* Ras-related G protein Rab-2-B (XP_015626284.1), OsYchF1 (BAD03576.1), *Arabidopsis thaliana* AtYchF1 (NP_174346.1), *Physcomitrella patens* YchF (XP_001776504.1), *Sorghum bicolor* YchF (XP_002445191.1), *Vitis vinifera* YchF (XP_002263885.1), *Capsicum annuum* YchF (AAF65513.1), Ricinus communis YchF (XP_002523184.1), and *Picea sitchensis* YchF (ABK25108.1) were analyzed using Jalview version 2.11.1.3 according to neighbor joining using amino acid (%) identity.

To extrapolate the features to all plants and animals, the G1-G5 motifs of the *R. norvegicus* heterotrimeric G protein α-subunit (PDB code: 1SVS) and small human Ras-related G protein C (PDB code: 3LLU) were compared with those of OsYchF1 in plants (PDB code: 5EE0). The G1 motif (phosphate binding loop) in OsYchF1 is highly identical to that of the *R. norvegicus* heterotrimeric G protein α-subunit and human Ras-related G protein C (Figures 2d-f and 3). Furthermore, the side-chain of the conserved Lys in the G4 motif ((N/T)**K**xD) of the *R. norvegicus* heterotrimeric G protein α-subunit and human Ras-related G protein C pointed inside the nucleotide-binding site and came into contact with ribose of guanosine nucleotide to support the GTP docking (Figure 2h and i). Oδ1/2 of Asp interacted with the amino group of guanosine, and the side chain of Asp formed a strong hydrogen bond with the amino group in the purine ring of guanosine (Figure 2h and i). In the YchF subfamily, however, the hydrophilic Lys residue was replaced by a group of hydrophobic amino acid residues, such as methionine (Met/M), valine (Val/V), or leucine (Leu/L) (Figure 3). The side chain of Met, Val, or Leu pointed outside of the nucleotide-binding site (Figure 2g). Therefore, the backbone of the carboxyl group of Met, Val, or Leu exposed inside and aided to form a hydrogen bond with the adenine 6-amino group properly. After site-directed mutagenesis in the G4 motif (N**M**S**E**) of OsYchF1, Met, and glutamic acid (Glu/E) were replaced by Lys and Asp, respectively. The OsYchF1 G4 motif mutant became GTP priority again.^10,11^ These results indicated that the G4 motif determined the ATP or GTP binding and hydrolysis specificity of OsYchF1.

To further illuminate the binding modes of G proteins with GTP or ATP, we selected four G protein complexes as examples, including *R. norvegicus* heterotrimeric G protein α-subunit with GMPPNP (guanylyl-imidodiphosphate) (PDB code: 1SVS), small human Ras-related G protein C with GMPPNP (PDB code: 3LLU), and OsYchF1 in the presence of GMPPNP (PDB code: 5EE9) or AMPPNP (adenylyl-imido-diphosphate) (PDB code: 5EE3), and conducted all-atomic molecular dynamics (MD) simulations. Either GTP or ATP binds to G proteins tightly, and the disassociation of G protein complexes was not observed during MD simulations. Yet, each complex showed specific interactions.

First, for 1SVS, GTP was completely surrounded by the protein. There were on average 5.3 hydrogen bonds between 1SVS and GTP. Two key serine residues of the protein, S16 and S120, formed critical hydrogen bonds with the small molecule (Figure 5a).

**Figure 5.**
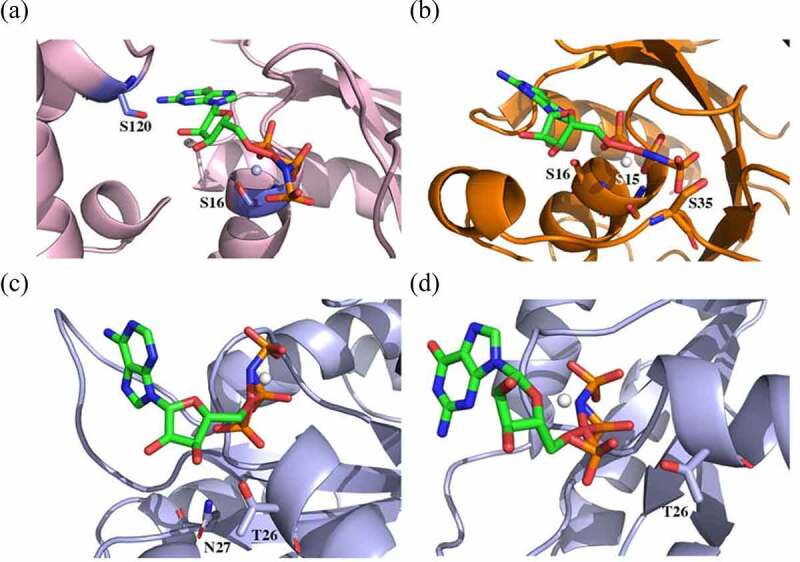
Snapshots of MD simulations illustrating the interaction in selected G protein complexes. (a) R. norvegicus heterotrimeric G protein α-subunit with GMPPNP (guanylyl imidodiphosphate) (1SVS), (b) small human Ras-related G protein C with GMPPNP (3LLU), and (c and d) OsYchF1 in the presence of GMPPNP and AMPPNP (adenylyl-imidodiphosphate) (5EE9 and 5EE3).

Second, although GTP partially exposed to the solution, more hydrogen bonds (11.1) were observed between 3LLU and GTP. However, many of such hydrogen bonds involved G protein backbone atoms. Notably, the hydroxy groups from S15, S16, and S35 sidechains hydrogen-bonded to GTP (Figure 5b).

Third, OsYchF1 with GMPPNP (PDB code: 5EE9) and AMPPNP (PDB code: 5EE3) was structurally similar, and therefore, their complexes resembled each other. It was worth noting that the whole ligand molecules were attached to OsYchF1 in crystal structures of both complexes. Interestingly, GTP and ATP exhibited high flexibility in conformation during MD simulations. Only triphosphate groups were anchored upon the unconventional G protein OsYchF1, while either guanine or adenine groups were solvated by water molecules. We speculated that there existed strong electrostatic interactions regarding triphosphate groups. Such a binding mode resulted in less hydrogen bonds (6.0 for the 5EE3 complex and 5.5 for the 5EE9 complex, respectively). We identified that T26 was essential for GTP and ATP binding modes (Figure 5c). In both complexes, hydrogen bonds were observed with respect to this residue. Noticeably, N207 also forms a hydrogen bond with AMPPNP in 5EE3, contributing to the stability of the complex, while such interaction was missing in the 5EE9 complex (Figure 5d).

Although the 3-D structure of OsYchF1 in the absence or presence of AMPPNP and GMPPNP had been determined by X-ray crystallography, the biological function of *OsYchF1* in rice is still not fully understood. Especially, the *OsYchF1* loss-of-function mutants and gain-of-function transgenic lines have not been constructed and observed in rice, and the important agronomic traits of *OsYchF1* need to be clarified in rice.
